# Revision and psychometric properties of the negative cognitive processing bias scale

**DOI:** 10.3389/fpsyt.2022.1013108

**Published:** 2022-11-03

**Authors:** Kuan Miao, Xuerong Liu, Xiaoling Zhang, Yuanyuan Li, Xingya Liao, Rui Zhang, Zhengzhi Feng, Zhiyi Chen

**Affiliations:** ^1^School of Psychology, Army Medical University, Chongqing, China; ^2^Western Medical Branch of PLA General Hospital, Beijing, China; ^3^School of Psychology, Experimental Research Center for Medical and Psychological Science (ERC-MPS), Army Medical University, Chongqing, China

**Keywords:** negative cognitive processing bias, mental health, depression, scale revision, psychometric properties, measurement

## Abstract

Negative cognitive processing bias (NCPB) is a cognitive trait that makes individuals more inclined to prioritize negative external stimuli (cues) when processing information. Cognitive biases have long been observed in mood and anxiety disorders, improving validation of tools to measure this phenomenon will aid us to determine whether there is a robust relationship between NCPB and major depressive disorder, anxiety disorders and other clinical disorders. Despite the development of an initial measure of this trait, that is, the negative cognitive processing bias questionnaire (NCPBQ), the lack of psychometric examinations and applications in large-scale samples hinders the determination of its reliability and validity and further limits our understanding of how to measure the NCPB traits of individuals accurately. To address these issues, the current study evaluated the psychometric properties of the NCPBQ in a large-scale sample (*n* = 6,069), which was divided into two subsamples (Subsample 1, *n* = 3,035, serving as the exploratory subsample, and Subsample 2, *n* = 3,034, serving as the validation subsample), and further revised it into a standardized scale, that is the negative cognitive processing bias scale (NCPBS), based on psychometric constructs. The results show that NCPBS possesses good construct reliability, internally consistent reliability, and test-retest reliability. Furthermore, by removing two original items from NCPBQ, NCPBS was found to have good criterion-related validity. In conclusion, the present study provides a reliable and valid scale for assessing negative cognitive processing bias of individuals.

## Introduction

Negative cognitive processing bias (NCPB) is a cognitive trait which not only directs attention to negative internal or external stimuli but also leads to misinterpretation of this information in a more negative way (1). Thus, the negative impacts that NCPB may have on psychological health and psychiatric conditions have sparked great interest in the scientific community. For instance, a study has demonstrated the prominent predictive role of NCPB on short-sighted judgments and decisions (2). Furthermore, NCPB has been revealed as one of the most common phenomena of major depressive disorder (MDD) and anxiety disorders (3, 4). Moreover, NCPB has been established to not only play an important role in the onset of depression, but also maintain depressed mood states (5). To reveal why a close association between NCPB and these mood disorders exists, Beck (6) explained that hypersensitivity in processing negative information (i.e., NCPB) in daily life is the key cognitive pedestal for depression symptom development and maintenance (6). Numerous studies have shown a robust link between NCPB and cognitive-related mental health problems (7, 8). Although NCPB is important for mental health, reliable and valid tools to measure this trait accurately are still scarce.

Different aspects of NCPB in depression and anxiety have been examined, including attention bias (9), memory bias (10), and interpretation bias (11, 12). Negative attention bias, which acts as the first filter for information selection, shows an attentional preference for negative stimuli and deviation from positive stimuli (13). Negative memory bias is the inclination to recall negative materials more often than positive materials. A supporting evidence is that patients with MDD show poorer recall performance for positive stimuli in memory tests than healthy controls (14). Moreover, interpretation bias involves prominent preferences for interpreting information or materials in negative ways (15). Recently, the response styles theory (RST) proposed a new framework for explaining NCPB for repetitive rethinking of negative memories and information that predominates transdiagnostic hallmark across mood disorders (16). Thus, predominating negative information in repetitive rethinking – that is, rumination – has been increasingly indicated to be an additional profile for NCPB (17).

Despite the lack of a reliable and valid scale to measure NCPB in terms of the conceptual structure mentioned above, several tools have been developed to partly assess cognitive bias. For example, the Dysfunctional Attitudes Scale (DAS) (18) was built to assess individuals’ maladaptive attitudes and beliefs about life and contains three main facets: perfectionism, utilitarianism, and criticism, which are not fully equal to one’s cognitive traits (i.e., NCPB). In addition, the Automatic Thoughts Questionnaire (ATQ) (19) was developed to investigate the frequency of negative automatic thoughts in self-statements. However, it focuses on individuals’ automatic thoughts with multiple cognitive components rather than cognitive traits. Furthermore, the Cognitive Bias Questionnaire (CBQ) (20) and Cognitive Style Questionnaire (CSQ) (21) were developed to measure one’s cognitive processing styles, such as negative self-evaluation, cognitive distortion, and outcome expectation. However, both questionnaires aim to measure one’s daily life behaviors and quantify the NCPB trait indirectly. Recently, the Negative Cognitive Processing Bias Questionnaire (NCPBQ) (22) was initially proposed to measure NCPB directly following four subdimensions. However, due to the lack of psychometric examinations and limited applications in small-scale samples, such as military personnel (23) and elderly individuals (24), the reliability and validity of the NCPBQ remain unclear. Furthermore, disparities in the construct structure of NCPBQ were found in previous studies (23, 24). Thus, it is necessary to examine the psychometric properties of NCPBQ and revise it into a standardized scale.

To address these issues, we recruited a large-scale sample (*n* = 6,069) across mainland China. In Subsample 1 (*n* = 3,035), we examined the reliability of the original version of NCPBQ using internal consistency analysis and test-retest analysis. In addition, validity was examined using criterion-related analysis and exploratory factor analysis (EFA) model. Furthermore, the NCPBQ was revised using standardized pipelines for building the NCPB scale. Finally, confirmatory factor analysis (CFA) was used for construct validity examination in Subsample 2 (*n* = 3,034).

## Materials and methods

### Participants

A large-scale sample (*n* = 6,069) was recruited using a hierarchical random sampling method (45.18% females and a mean age of 31.44 years, SD = 8.99, range = 18–65 years). Participants were recruited based on the provincial population distribution in mainland China, with a larger number of participants recruited in provinces with a larger population (e.g., Guangdong, Shandong and Henan). This sample pool covered the vast majority of occupations in mainland China (e.g., students, farmers, and businessmen). The sociodemographic features and results of statistical test are shown in Table 1.

**TABLE 1:** Descriptive characteristics of the participants (*n* = 6,069).

Variables	Grouping	Frequency	Percent (%)	χ^2^	*p*
Gender	Female	2,736	45.18	58.73	0.000***
	Male	3,333	54.82		
Age	18–25	1,578	26.00	3,967.95	0.000***
	26–35	2,879	47.44		
	36–45	1,109	18.27		
	46–55	406	6.69		
	56–65	97	1.60		
Educational attainment	Primary school	305	5.03	6,092.57	0.000***
	Middle school	497	8.19		
	High school	1,344	22.15		
	Bachelor’s degree	3,530	58.16		
	Master’s degree or above	393	6.47		
Occupation	Student	711	11.72	3,580.89	0.000***
	Farmer	379	6.24		
	Manual worker	648	10.68		
	Military personnel	346	5.70		
	Public servant	1,070	17.63		
	Businessman/Office worker	2,402	39.58		
	Intellectual/Scientific researcher	513	8.45		

***p < 0.001.

All the participants were instructed to complete an online survey *via* a webpage and received payment for their participation. All the participants provided written informed consent preceding access to the online questionnaire. In addition, items for lie detection were included in the survey for quality control. A third-party platform (WJX Platform, Ran-Xin Technique Co. Ltd., Changsha, China) was involved in the sampling, data acquisition and quality control. This study was approved by the IRB of Army Medical University (China).

### Measures

#### Sociodemographic characteristics

To ensure sample representativeness, a sociodemographic investigation was conducted. This part included items as follows: gender, age, educational attainment, and occupation.

#### Negative cognitive processing bias questionnaire

The NCPBQ was initially developed by Yan et al. (22) to assess cognitive processing traits. The original version of the NCPBQ contained 23 self-reported items rated on a 4-point Likert-scale style (“1” for “never”; “4” for “always”) in four dimensions: negative attention bias (NAB, e.g., My attention is easily drawn to the tragic images on TV and is difficult to shift.), negative memory bias (NMB, e.g., I can easily remember the negative comments people make about me.), negative interpretation bias (NIB, e.g., If a new leader or teacher is hard on me, I think it is because he sees me in a bad light and wants to get me in trouble.), and negative rumination bias (NRB, e.g., I often think about why I am so sad). Each dimension included five items, except these, there are three lie detection items in the original measure (items 4, 16, and 23. e.g., I have never told a lie.).

#### Dysfunctional attitude scale

The DAS consists of 40 items evaluating respondents’ attitudes toward daily life, such as “undesirable life attitudes or beliefs,” “black-and-white attitudes for moral judgment” and “perfectionism.” This measure uses a 7-point Likert scale ranging from “completely disagree” to “completely agree,” with higher scores indicating more maladaptive attitudes. This scale has been found to have high reliability (Cronbach’s α = 0.93) in psychometric examinations (25).

#### Beck depression inventory II

Developed and revised by Beck (26), Beck Depression Inventory II (BDI-II) contains 21 self-reported items and has long been acknowledged as one of the most broadly certified tools for assessing the severity of MDD (27). This inventory has been validated for good reliability and validity in clinical practice (28). The internal and test-retest reliability of the BDI-II of the Chinese version was found to be good (Cronbach’s α = 0.94; *r*_*test–retest*_ = 0.55) (29).

### Statistical analyses

The full sample was divided into two subsamples, with one serving as an exploratory subsample (Subsample 1, *n* = 3,035) and the other serving as the validation subsample (Subsample 2, *n* = 3,034). Descriptive statistics were first reported for both subsamples. Item analysis was performed to examine the items’ suitability. Furthermore, to reveal the factor structures of the original version of the NCPBQ, EFA was conducted with principal component analysis (PCA), dimension reduction and varimax rotation in Subsample 1. By visual inspection of the scree plot and psychometric criteria (factor eigenvalues > 1.0), the number of factor structures was determined. Moreover, to revise the original version of the NCPBQ for a better factor structure, items with loadings under 0.50 were removed (30). Finally, CFA was carried out on the revised negative cognitive processing bias scale (NCPBS) in Subsample 2 to validate the factor structure. Seven metrics assessing goodness-of-fit were drawn to evaluate this model, including root mean square error of approximation (RMSEA), standardized root mean square residual (SRMR), goodness-of-fit index (GFI), comparative fit index (CFI), normed fit index (NFI), incremental fit index (IFI), and the Tucker-Lewis Index (TLI). An RMSEA and SRMR of <0.05; a GFI and CFI > 0.95; and a NFI, IFI, and TLI > 0.90, together, would suggest a good model fit (31).

The reliability was evaluated mainly *via* internal consistency reliability and test-retest reliability. Cronbach’s alpha (α) and McDonald’s omega (ω) were used to measure the internal consistency reliability. Since there is an argument regarding which is the best measure for assessing internal consistency reliability (32), α and ω were both calculated, with values of 0.70 or higher considered acceptable (33). In addition to internal consistency reliability, the 2-month test-retest reliability was also evaluated. A Pearson *r* value >0.50 indicated good test-retest reliability for a given scale (34).

The criterion-related validity of the revised NCPBS was estimated by the Pearson bivariate correlations across the NCPBS, BDI-II, and DAS, with significantly positive correlations between the NCPBS and both the BDI-II and DAS for high validity.

To gain further insights into the validity of this revised scale, between-group differences were examined for demographic features, which were compared by using independent sample *t*-tests or one-way ANOVA (Bonferroni correction for *post hoc* test), including gender, age, and education. The participants were classified into three age groups: early-adult group (aged 18–30), mid-adult group (aged 31–45), and old-adult group (aged 46–65). Additionally, education level was divided into two group: well-educated group for educational experiences >13 years and a less-educated group for educational experiences <13 years.

The data were analyzed by IBM SPSS Statistics 26.0 (Armonk, NY: IBM Corp.), Amos 21.0 programs, and JASP 0.16.2.^1^

## Results

### Descriptive statistics

The descriptive statistics for each item and subdimensions were tabulated (see Table 2), including the mean value, standard deviation and normality (estimated by Kolmogorov–Smirnov test, skewness, and kurtosis). Although, the results showed each item failed the Kolmogorov–Smirnov test, we found no prominent irregular skewness or kurtosis for this sample (The distribution of item scores was listed in Supplementary Figures). Furthermore, a similar pattern was found in each dimension, including negative attention bias (NAB, 11.47 ± 3.06 for items 1, 6, 13, 17, and 21), negative memory bias (NMB, 10.60 ± 2.67, for items 2, 8, 11, 15, and 20), negative interpretation bias (NIB, 11.21 ± 2.87, for item 3, 5, 9, 12, and 18), and negative rumination bias (NRB, 8.66 ± 2.71, for items 7, 10, 14, 19, and 22).

**TABLE 2:** Descriptive statistics and the normality of data.

Item	Item score		Kolmogorov–Smirnov test		Skewness and kurtosis	
	*M*	SD	*D*-value	*P*-value	*S*	*K*
P1	2.48	0.782	0.249	0.000***	–0.075	–0.425
P2	2.59	0.938	0.205	0.000***	–0.043	–0.897
P3	2.15	0.864	0.244	0.000***	0.334	–0.581
P5	2.07	0.759	0.279	0.000***	0.345	–0.206
P6	2.26	0.850	0.235	0.000***	0.159	–0.645
P7	2.42	0.913	0.214	0.000***	0.064	–0.808
P8	2.78	0.848	0.258	0.000***	–0.295	–0.504
P9	2.33	0.867	0.250	0.000***	0.221	–0.602
P10	2.12	0.963	0.223	0.000***	0.451	–0.790
P11	2.68	0.922	0.229	0.000***	–0.200	–0.797
P12	2.43	0.864	0.227	0.000***	0.063	–0.659
P13	2.10	0.860	0.258	0.000***	0.454	–0.417
P14	2.41	0.926	0.209	0.000***	0.070	–0.853
P15	2.60	0.939	0.220	0.000***	–0.128	–0.870
P17	2.24	0.799	0.258	0.000***	0.175	–0.465
P18	2.23	0.867	0.252	0.000***	0.298	–0.566
P19	2.12	0.897	0.239	0.000***	0.406	–0.620
P20	2.74	0.870	0.257	0.000***	–0.296	–0.561
P21	2.39	0.915	0.218	0.000***	0.100	–0.811
P22	2.01	0.856	0.247	0.000***	0.516	–0.397
NAB	11.47	3.056	0.086	0.000***	0.108	–0.403
NMB	10.601	2.674	0.081	0.000***	–0.119	–0.443
NIB	11.206	2.870	0.087	0.000***	0.177	–0.240
NRB	8.66	2.712	0.103	0.000***	0.285	–0.451
Total	41.94	8.767	0.035	0.000***	–0.051	–0.092

M, mean; SD, standard deviation; NAB, negative attention bias; NMB, negative memory bias; NIB, negative interpretation bias; NRB, negative rumination bias.

***p < 0.001.

### Item analysis

To examine the validity of each item, critical ratio (CR) method, Pearson correlation method and a homogeneity test were applied to Subsample 1 for item analysis. The results revealed significant score differences between the high-total-score (top 27%) and low-total-score groups (last 27%) for all items, irrespective of CR values, indicating that all the items possessed high discrimination power (see Table 3). Further analysis also illustrated statistically significant item-total correlations. Finally, the results of the homogeneity test found acceptable communalities and factor loadings, except for item 8 (see Table 3, communalities = 0.178, factor loading = 0.422). As a result, item 8 (I always remember my mistakes clearly) was removed in this step.

**TABLE 3:** Results of the item analysis.

	Extreme groups analysis	Item-total correlation		Homogeneity test		
Item	Critical ratio value	Item-total correlation	Corrected item-total correlations	Cronbach’s α if item omitted	Communalities	Factor loading
P1	41.284***	0.519***	0.456	0.873	0.278	0.527
P2	46.056***	0.553***	0.480	0.872	0.299	0.547
P3	39.364***	0.501***	0.429	0.874	0.247	0.497
P5	39.606***	0.504***	0.441	0.873	0.258	0.508
P6	45.844***	0.553***	0.487	0.872	0.315	0.561
P7	52.139***	0.593***	0.526	0.870	0.350	0.592
P8	33.026***	0.439***	0.364	0.876	0.178	0.422
P9	49.757***	0.588***	0.524	0.871	0.350	0.592
P10	47.672***	0.564***	0.490	0.872	0.313	0.559
P11	50.289***	0.585***	0.517	0.871	0.340	0.583
P12	40.065***	0.508***	0.437	0.873	0.253	0.503
P13	42.641***	0.539***	0.471	0.872	0.298	0.546
P14	38.725***	0.501***	0.423	0.874	0.238	0.488
P15	46.622***	0.567***	0.495	0.871	0.316	0.562
P17	47.257***	0.582***	0.523	0.871	0.355	0.596
P18	42.299***	0.517***	0.447	0.873	0.264	0.514
P19	49.821***	0.590***	0.524	0.870	0.352	0.593
P20	49.701***	0.598***	0.535	0.870	0.359	0.599
P21	46.778***	0.568***	0.498	0.871	0.328	0.573
P22	52.035***	0.601***	0.539	0.870	0.368	0.607

***p < 0.001.

### Exploratory factor analysis

The results demonstrated the suitability of EFA for the current dataset with an acceptable KMO coefficient (=0.93) and significant skewness from a spherical distribution (χ^2^ = 15,492.751, *p* < 0.001). Furthermore, principal axis factoring (PAF) was adopted for factor extraction and loading estimation, with <0.5 used as the exclusion criterion. The results indicated a four-dimensional structure for the NCPBQ, in which four common factors with eigenvalues >1 in orthogonal rotation from the maximum variance method), accounting for 52.87% of the total variance (see Table 4). Although the four-facet construct structure was validated here, item 7 was found to be unacceptable, as its factor loading was less than 0.50 (factor loading = 0.492). Finally, item 7 (I often think about why I am always inferior to others) was removed in this step.

**TABLE 4:** Standardized factor loading of the negative cognitive processing bias scale.

Item		NAB	NMB	NIB	NRB
1	My attention is easily drawn to tragic images on TV and is difficult to shift	0.728			
17	My attention is easily drawn to the sad expressions of others and is difficult to shift	0.719			
6	My attention is easily drawn to harrowing sounds and is difficult to shift	0.690			
21	My attention is easily drawn to the tragic storylines of the novels and is difficult to shift	0.653			
13	My attention is easily drawn to the hesitant eyes of others and is difficult to shift	0.602			
20	I can easily remember the negative comments people make about me		0.731		
15	Even if I think I have done nothing wrong, I remember the criticism of others for a long time		0.711		
11	In the process of interacting with others, if I say the wrong thing, I will not forget it for a long time		0.665		
2	I still vividly remember a time when I was ridiculed		0.562		
5	If I meet a friend for the first time and he (she) says very little to me, I will think he or she doesn’t like me			0.730	
3	If an acquaintance walks across the street and does not say hello to me, I will think he or she has a problem with me			0.643	
18	If a new leader or teacher is hard on me, I think it is because he sees me in a bad light and wants to get me in trouble			0.633	
9	If I were to go on stage and give a speech in public, and when I come down, I see a few people next to me whispering, I think they are laughing at my bad speech			0.594	
12	If I participated in a job applications and the interviewer had a serious expression throughout the process, I would think that the application would most likely fail.			0.525	
22	I often think about why I am so sad				0.702
19	I often think about why my mood is low and those of others are not				0.701
10	I often think about why I am so lonely				0.675
14	I often think about why I lack interest and motivation to do things				0.652
Percent of variance		14.787%	12.171%	13.017%	12.894%

NAB, negative attention bias; NMB, negative memory bias; NIB, negative interpretation bias; NRB, negative rumination bias.

On balance, the original version of the NCPBQ has been revised by removing two items (item 7 and item 8) based on the above results.

### Validity analysis

#### Confirmatory factor analysis

**FIGURE 1:**
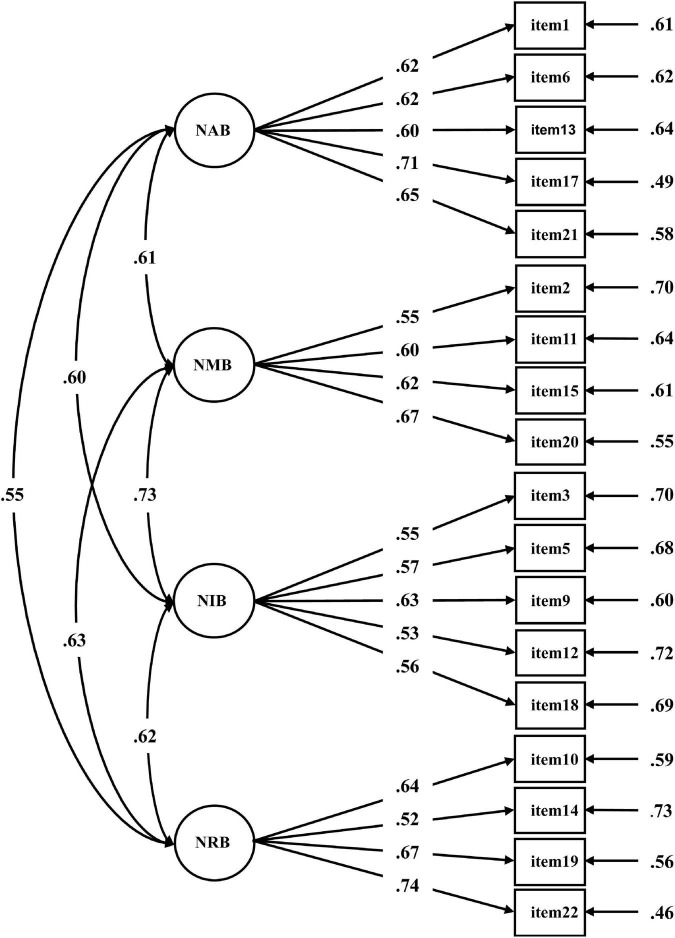
The four-factor confirmatory factor analysis model of the negative cognitive processing bias scale for the validation set (*n* = 3,034). Each number alongside the lines indicates standardized factor loading. NAB, negative attention bias; NMB, negative memory bias; NIB, negative interpretation bias; NRB, negative rumination bias.

To estimate the construct validity for the revised version, CFA was carried on this four-dimensional structure in independent Subsample 2. The results revealed good goodness-of-fit metrics for the revised NCPBS (RMSEA = 0.04, SRMR = 0.03, and GFI = 0.97, more details in Table 5). In addition, all items were found to have acceptable factor loadings (β = 0.52–0.74, see Figure 1).

**TABLE 5:** Confirmatory factor analysis model fit indexes.

	RMSEA	SRMR	GFI	CFI	NFI	IFI	TLI
Criteria	<0.05	<0.05	>0.95	>0.95	>0.90	>0.90	>0.90
Fit indexes	0.04	0.03	0.97	0.96	0.95	0.96	0.95

RMSEA, root mean square error of approximation; SRMR, standardized root mean square residual; GFI, goodness-of-fit index; CFI, comparative fit index; NFI, normed fit index; IFI, incremental fit index; TLI, Tucker-Lewis Index.

#### Criterion-related validity

To test the validity of the revised NCPBS, the criterion-related validity was estimated by correlating its scores to those of the DAS and BDI-II. The results showed that the scores for both DAS (*r* = 0.551, *p* < 0.001) and BDI-II (*r* = 0.447, *p* < 0.001) were significantly correlated with the total score of the revised NCPBS and even its subdimensions (see Table 6).

**TABLE 6:** Pearson’s correlations between the negative cognitive processing bias scale and the criterion measures.

	Overall scale	NAB	NMB	NIB	NRB
DAS	0.551***	0.357***	0.376***	0.494***	0.466***
BDI-II	0.447***	0.315***	0.346***	0.328***	0.377***

NAB, negative attention bias; NMB, negative memory bias; NIB, negative interpretation bias; NRB, negative rumination bias.

***p < 0.001.

### Reliability analysis

Cronbach’s α and McDonald’s ω were calculated to estimate the internal consistency of the revised NCPBS. The results showed good internal consistency reliability for this revised version, including the whole scale and its subdimensions (both α and ω = 0.866, see Table 7). Furthermore, significant correlations were found in the 2-month test-retest consistency analysis, demonstrating good test-retest consistency reliability for the revised NCPBS (*r* = 0.943 for overall scale scores, more results can be found in Table 7).

**TABLE 7:** Cronbach’s α, McDonald’s ω, and test-retest reliability of the negative cognitive processing bias scale.

	Cronbach’s *a*	McDonald’s ω	Test-retest reliability
Overall scale	0.866	0.866	0.943
NAB	0.733	0.734	0.705
NMB	0.698	0.701	0.785
NIB	0.703	0.704	0.761
NRB	0.732	0.735	0.748

NAB, negative attention bias; NMB, negative memory bias; NIB, negative interpretation bias; NRB, negative rumination bias.

### Differential analysis for sociodemographic features

To examine whether the revised NCPBS was valid, differential analysis was conducted for sociodemographic features in the whole sample. The results revealed significant differences between genders for NCPBS scores (total for males: 41.34 ± 8.84, total for females: 42.67 ± 8.62, *t* = –5.936, *p* < 0.001; BF_10_ = 1.18 × 10^6^ at JSY Cauchy distribution, see Table 8). Furthermore, we also found difference between educational levels in the NCPBS scores, with low scores for low educational level (total for less-educated group: 39.80 ± 8.49, total for well-educated group: 43.10 ± 8.70, *t* = –14.258, *p* < 0.001; BF_10_ = 6.26 × 10^41^ at JSY Cauchy distribution, see Table 9). Finally, the NCPBS scores varied between age-related groups (total for early-adult group: 42.83 ± 8.96, total for mid-adult group: 40.97 ± 8.43, total for old-adult group: 40.53 ± 8.32, *F* = 38.082, *p* < 0.001, see Table 10).

**TABLE 8:** Results for gender differences in negative cognitive processing bias scale scores.

	Male (*n* = 3,333)	Female (*n* = 2,736)	*T*-value	*P*-value	BF_10_
NAB	11.25 ± 3.06	11.75 ± 3.03	–6.348	0.000***	1.458 × 10^6^
NMB	10.50 ± 2.67	10.72 ± 2.68	–3.239	0.001**	5.422
NIB	11.01 ± 2.88	11.45 ± 2.84	–6.027	0.000***	2.034 × 10^6^
NRB	8.58 ± 2.74	8.75 ± 2.68	–2.455	0.014*	0.586
Total	41.34 ± 8.84	42.67 ± 8.62	–5.936	0.000***	1.184 × 10^6^

BF, Bayesian factor; NAB, negative attention bias; NMB, negative memory bias; NIB, negative interpretation bias; NRB, negative rumination bias.

*p < 0.05, **p < 0.01, ***p < 0.001.

**TABLE 9:** Results for education level differences in negative cognitive processing bias scale scores.

	Less-educated (*n* = 2,146)	Well-educated (*n* = 3,904)	*T*-value	*P*-value	BF_10_
NAB	10.86 ± 2.88	11.80 ± 3.10	–11.648	0.000***	3.505 × 10^27^
NMB	10.07 ± 2.57	10.89 ± 2.68	–11.586	0.000***	1.740 × 10^27^
NIB	10.48 ± 2.80	11.59 ± 2.83	–14.618	0.000***	4.400 × 10^43^
NRB	8.38 ± 2.59	8.81 ± 2.76	–5.925	0.000***	1.155 × 10^43^
Total	39.80 ± 8.49	43.10 ± 8.70	–14.258	0.000***	6.263 × 10^41^

BF, Bayesian factor; NAB, negative attention bias; NMB, negative memory bias; NIB, negative interpretation bias; NRB, negative rumination bias.

***p < 0.001.

**TABLE 10:** Results for age differences in negative cognitive processing bias scale scores.

	18–30 (*n* = 3,278)	31–45 (*n* = 2,288)	46–65 (*n* = 503)	ANOVA		*Post hoc* test
				*F*-value	*P*-value	*P*-value (corrected)
NAB	11.60 ± 3.10	11.31 ± 2.97	11.41 ± 3.11	6.400	0.002**	1–2:0.001** 1–3:0.566 2–3: 1.000
NMB	10.87 ± 2.72	10.32 ± 2.59	10.15 ± 2.50	36.622	0.000***	1–2:0.000*** 1–3:0.000*** 2–3:0.625
NIB	11.45 ± 2.91	10.97 ± 2.81	10.70 ± 2.71	26.956	0.000***	1–2:0.000*** 1–3:0.000*** 2–3:0.148
NRB	8.92 ± 2.76	8.37 ± 2.62	8.27 ± 2.62	33.795	0.000***	1–2:0.000*** 1–3:0.000*** 2–3: 1.000
Total	42.83 ± 8.96	40.97 ± 8.43	40.53 ± 8.32	38.082	0.000***	1–2:0.000*** 1–3:0.000*** 2–3:0.918

NAB, negative attention bias; NMB, negative memory bias; NIB, negative interpretation bias; NRB, negative rumination bias.

**p < 0.01, ***p < 0.001. Bold font in *post hoc* test indicates p < 0.05.

## Discussion

The purpose of this study was to evaluate the psychometric properties of the original version of the NCPBQ in a large sample and to revise it into a reliable and valid scale. As we expected, the results showed good four-dimensional construct validity and reliability for the revised NCPBS. Furthermore, small differences in NCPBS scores across sociodemographic features, including gender, educational level, and age, were found, with old less-educated males exhibiting low NCPB. On balance, the current study revealed the psychometric properties of the initial NCPBQ and further revised it into a reliable and valid scale for measuring individuals’ cognitive trait in negative information processing.

Here, a weakly skewed distribution was found in the item analysis for NCPBS, which seemed to align with general prevalence of cognition-biased mood disorder (e.g., MDD). The lifetime prevalence of these psychiatric conditions that reported in previous literature, such as depressive disorders (6.8%) and anxiety disorders (7.6%), were low in China (35, 36). Accordingly, we found no prominent irregular skewness or kurtosis for this sample by canonical criteria (i.e., skewness < 3; kurtosis < 8) (37). In this vein, this finding indicated that the NCPBS may be a valid tool for measuring cognitive processing trait.

Furthermore, the present study addressed a long-standing debate over the factor structure of NCPB. Both EFA and CFA revealed that the four-factor structure possessed good construct validity for the revised NCPBS, which strongly supports RST (16). The conventional theoretical basis for NCPB frames individuals’ cognitive biases in terms of fundamental cognitive components, such as attention, memory, and interpretation (38). However, it should be borne in mind that these cognitive faculties bias individual’s behaviors through “processing.” Thus, as a typical processing style, rumination functions to boost cognitive biases based on these cognitive components, which may determine the extent to which NCPB increases the risk of mood disorders (39). Thus, this study clarified the potential structure of NCPB by psychometric methods. In addition, NCPBS showed better reliability and validity than the previous version. Thus, the major goal of the current study was to provide a standardized NCPBS to accurately measure individuals’ negative cognitive trait.

In addition to revising the NCPBS, some between-group differences were also found. Although the differences were very small, the exploratory explanations would be inferred here. Firstly, a small gender difference in the NCPBS scores was observed, with slightly higher scores in females. This result may be supported by both theoretical and empirical evidence, as gender-related environment susceptibility theory proposes that females detect more subtle negative cues from daily life events and the environment due to genetic imprinting (40–42). In addition, previous studies have validated this theoretical model, showing increased neural activity and behavioral reactions to negative information in females compared with that of males (43, 44). Thus, in the current study, these indirect evidence may imply higher negative information susceptibility in females compared to males. Furthermore, we observed slightly lower NCPB for individuals with a less educational level, which is consistent with previous evidence. Daraei and Ghaderi (45) documented the association of a low education level with optimism and well-being (45). Besides, compared to elders, young adults exhibited a little higher NCPB as measured by the revised NCPBS. This could be explained well by the differences in their emotional regulation ability. Existing studies have revealed that, as predicted by emotional regulation ability (including regulation resource and regulation strategy), older adults exhibit better decision-making ability in both positive and negative emotional conditions than young adults (46–48). Thus, we inferred that such age-related effects in emotional processing may cause different NCPB for distinct age groups. Together, these evidences suggest that the current study may provide a valid and reliable measure to quantify individuals’ cognitive trait, with potentials for application in psychological and psychiatric domains.

## Limitations

Several limitations in the current study should be borne in mind before applying the NCPBS. Despite claiming it to be a robust predictor of depression, little is known about whether this scale can be used in clinical practice because no depression patients were recruited in the present study. Thus, a cohort study for investigating the association between depression and NCPB in clinical practice is needed in the future. In addition, this large-scale sample was taken only from the Chinese population, which hampers the cross-cultural generalizability of this scale. To address this issue, future studies should examine the reliability and validity of the scale by using a broad sample.

## Conclusion

The current study recruited a large-scale sample to validate the psychometric properties of the NCPBQ, and followed a standardized pipeline to revise the scale. The results show that NCPBS has better reliability and validity than the original version, with higher internal consistency reliability and construct validity. Furthermore, we found the statistical differences in NCPB across sociodemographic features by using NCPBS, which provided further evidence of external validity of this scale. Taken together, this study provides a reliable and valid measure to estimate individuals’ cognitive inclinations toward negative content accurately and advanced our understanding of the core components of NCPB.

## Data availability statement

The datasets presented in this study can be found in online repositories. The names of the repository/repositories and accession number(s) can be found below: https://osf.io/ucmw4/.

## Ethics statement

The studies involving human participants were reviewed and approved by the IRB of Army Medical University (China). The patients/participants provided their written informed consent to participate in this study.

## Author contributions

KM: writing – original draft and review, and editing and data curation. XUL and XZ: writing – review and editing. YL, XIL, and RZ: data curation. ZF and ZC: conceptualization. All authors contributed to the article and approved the submitted version.

## Footnotes

1
https://jasp-stats.org/
