# Supplementary material for: Revision and psychometric properties of the negative cognitive processing bias scale
Source: Front Psychiatry. 2022 Nov 3;13:1013108. doi: 10.3389/fpsyt.2022.1013108 (PMC9669056; doi:10.3389/fpsyt.2022.1013108)
Supplement: Supplementary file 1 [file Data_Sheet_1.pdf]

Supplementary Materials for

Revision and psychometric properties of the Negative Cognitive Processing Bias Scale

Kuan Miao[1], Xuerong Liu[1], Xiaoling Zhang[1], Yuanyuan Li[2], Xingya Liao[1], Rui Zhang[1], Zhengzhi Feng[1*], Zhiyi Chen[1*]

[1]School of Psychology, Army Medical University, Chongqing, China
[2]Western Medical Branch of PLA General Hospital, Beijing, China
[3]School of Psychology, Experimental Research Center for Medical and Psychological Science (ERC-MPS), Army Medical University

*** Correspondence:**
Corresponding Author
Zhengzhi Feng: fzz@tmmu.edu.cn Zhiyi Chen: chenzhiyi@email.swu.edu.cn

Contents
25 of 25 Supplementary Figures

Supplementary Figures

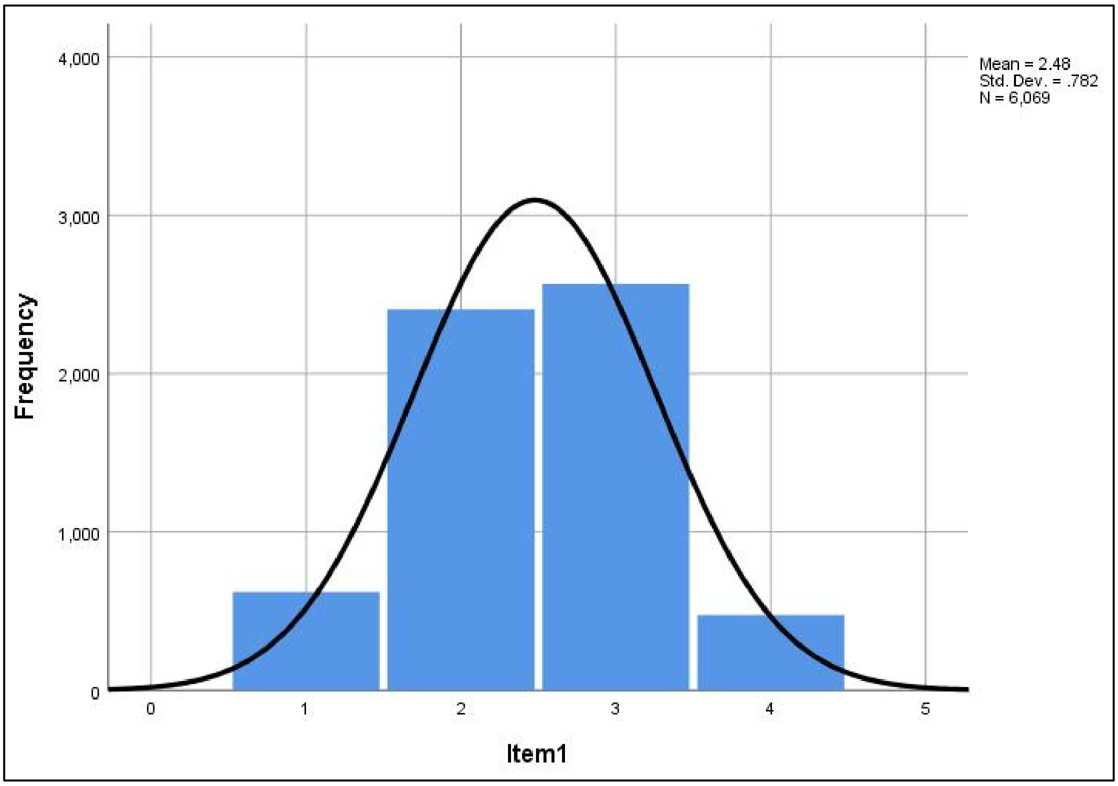

Figure S1 Distribution of scores in the item 1.

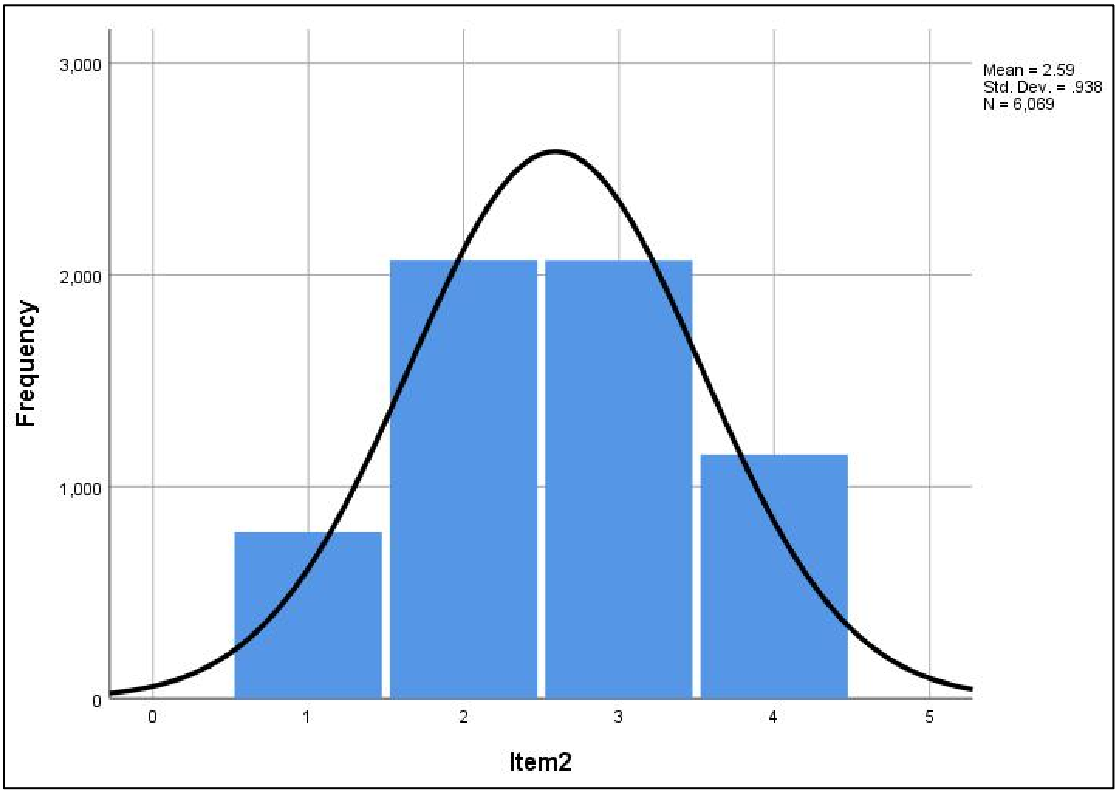

Figure S2 Distribution of scores in the item 2.

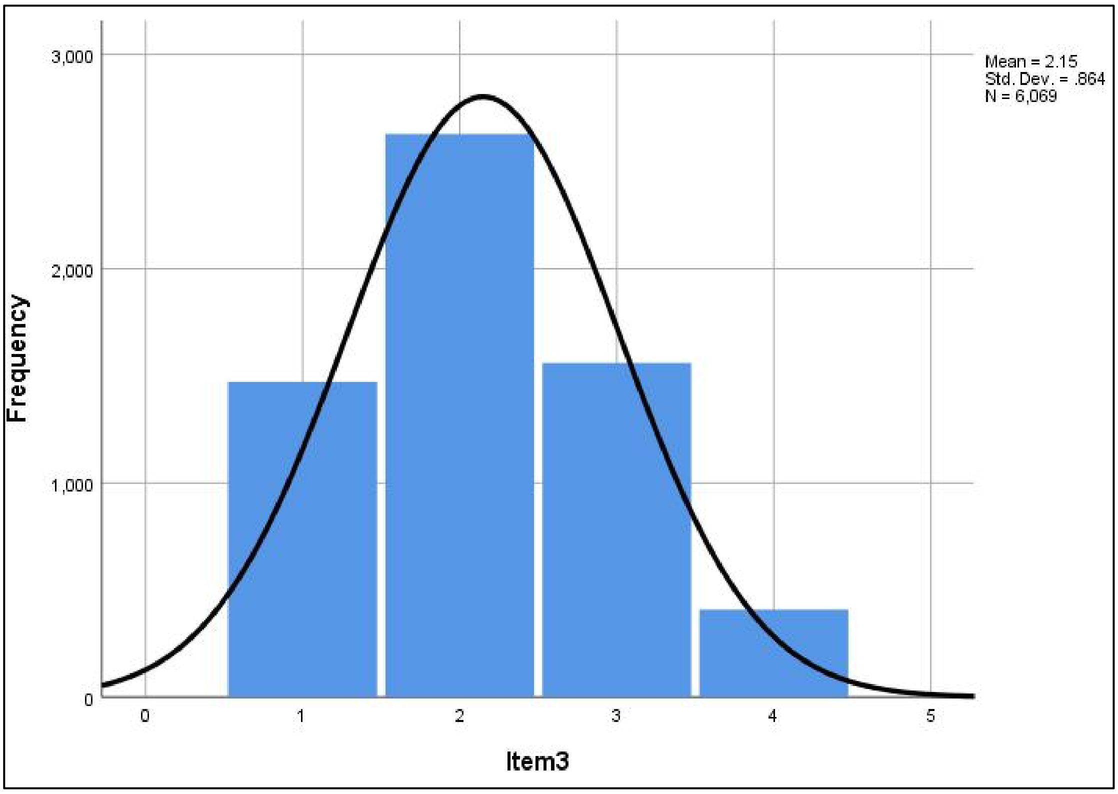

Figure S3 Distribution of scores in the item 3.

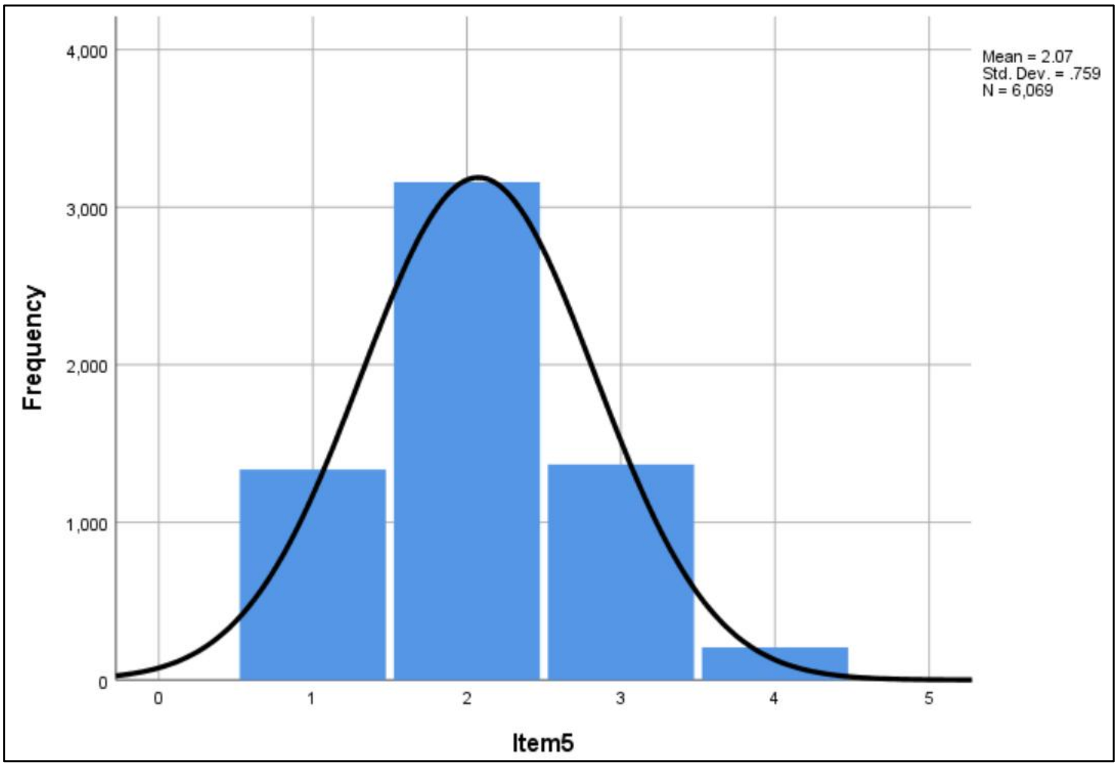

Figure S4 Distribution of scores in the item 5.

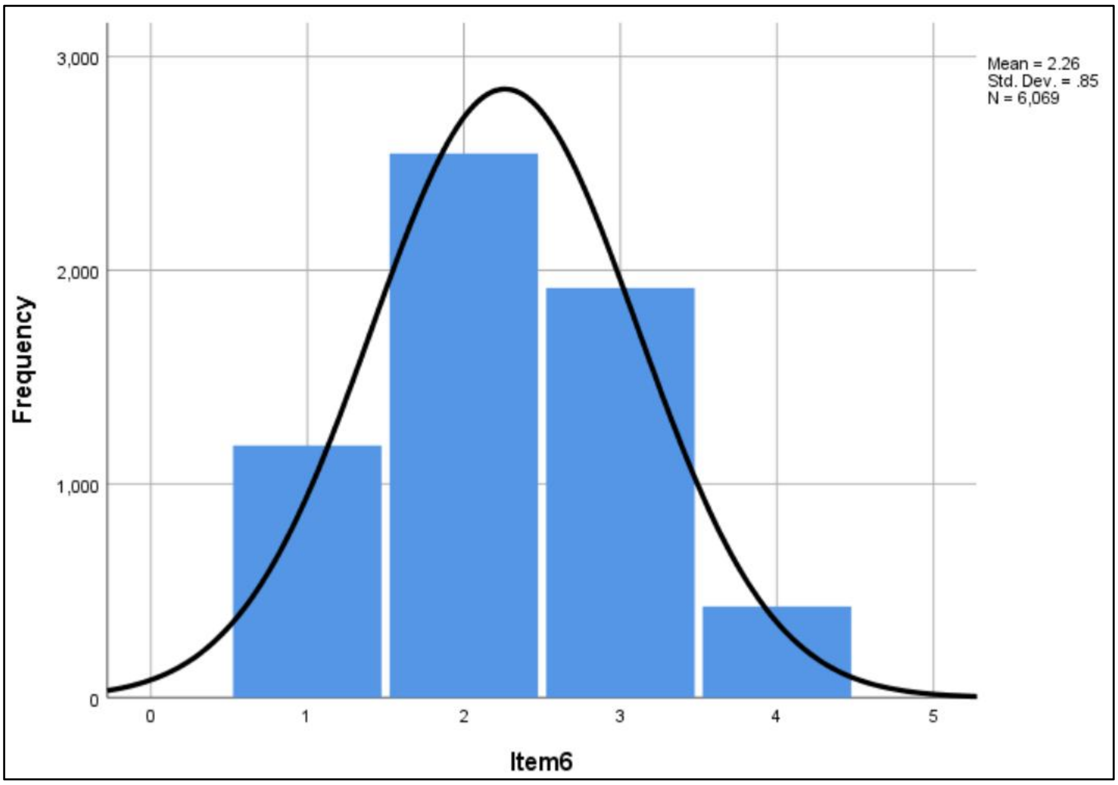

Figure S5 Distribution of scores in the item 6.

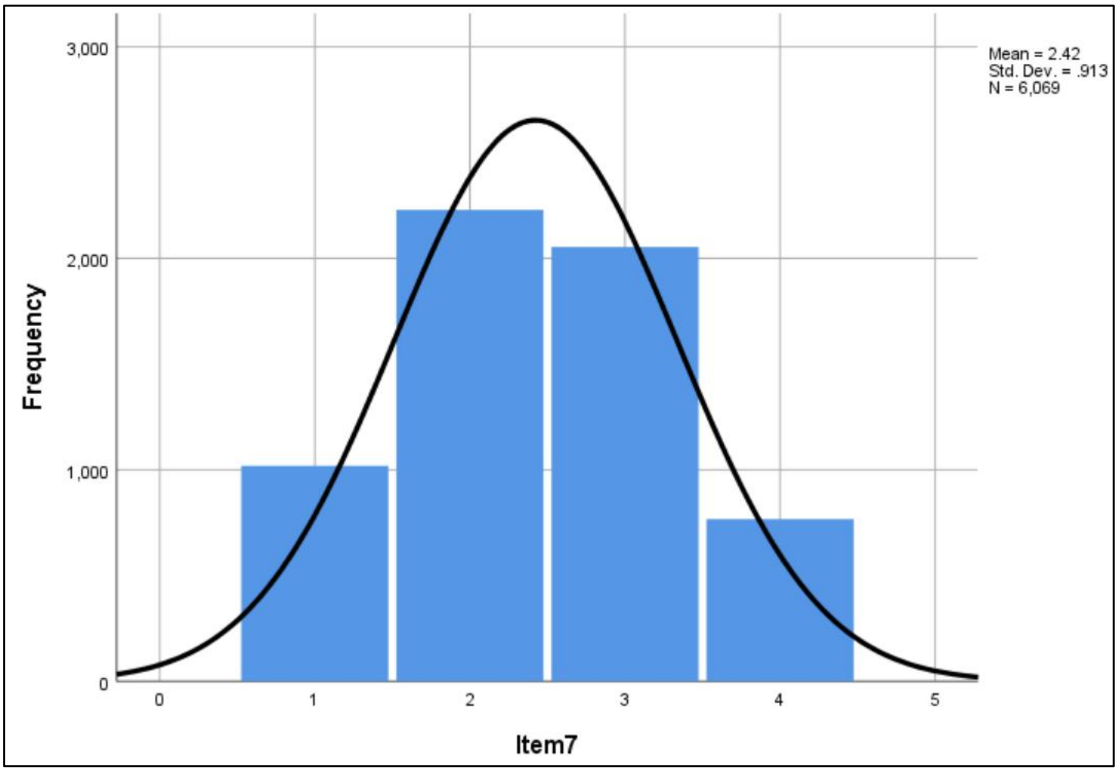

Figure S6 Distribution of scores in the item 7.

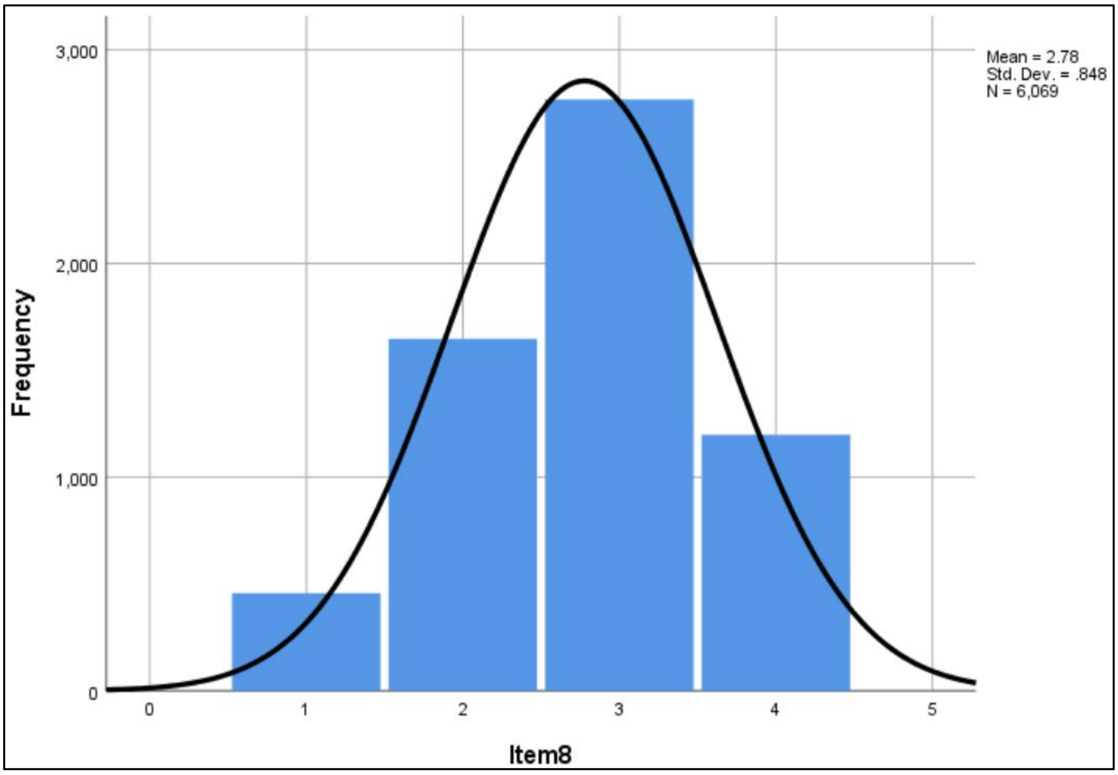

Figure S7 Distribution of scores in the item 8.

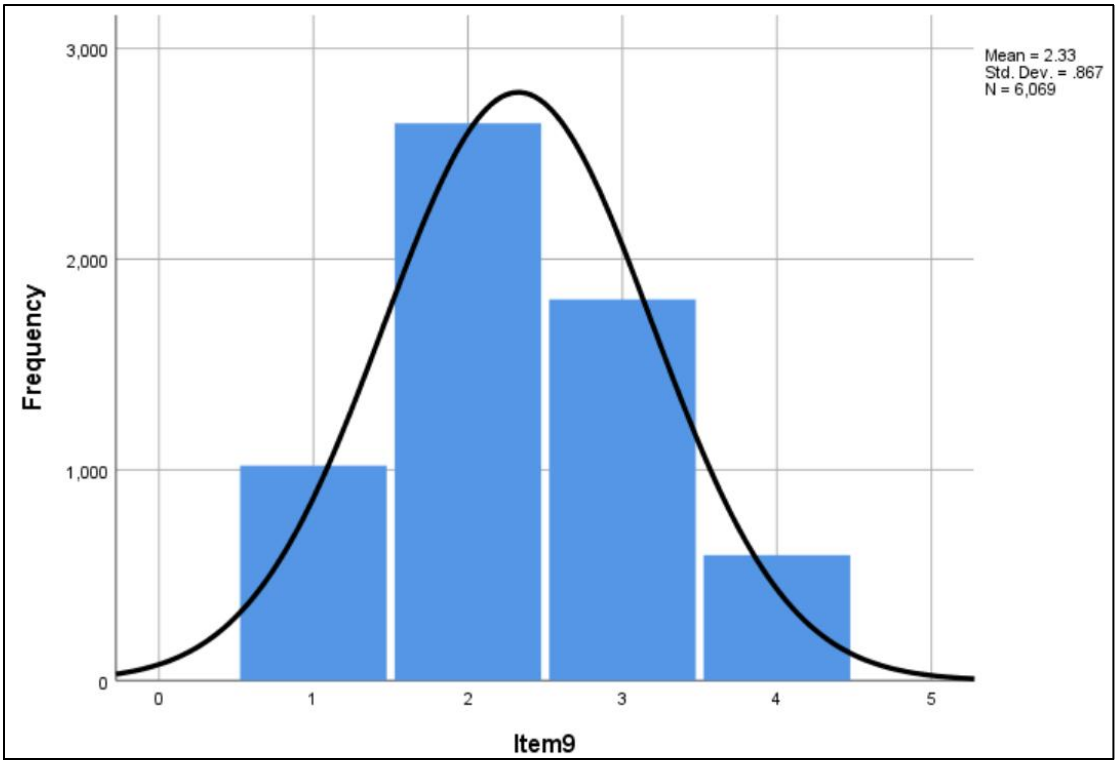

Figure S8 Distribution of scores in the item 9.

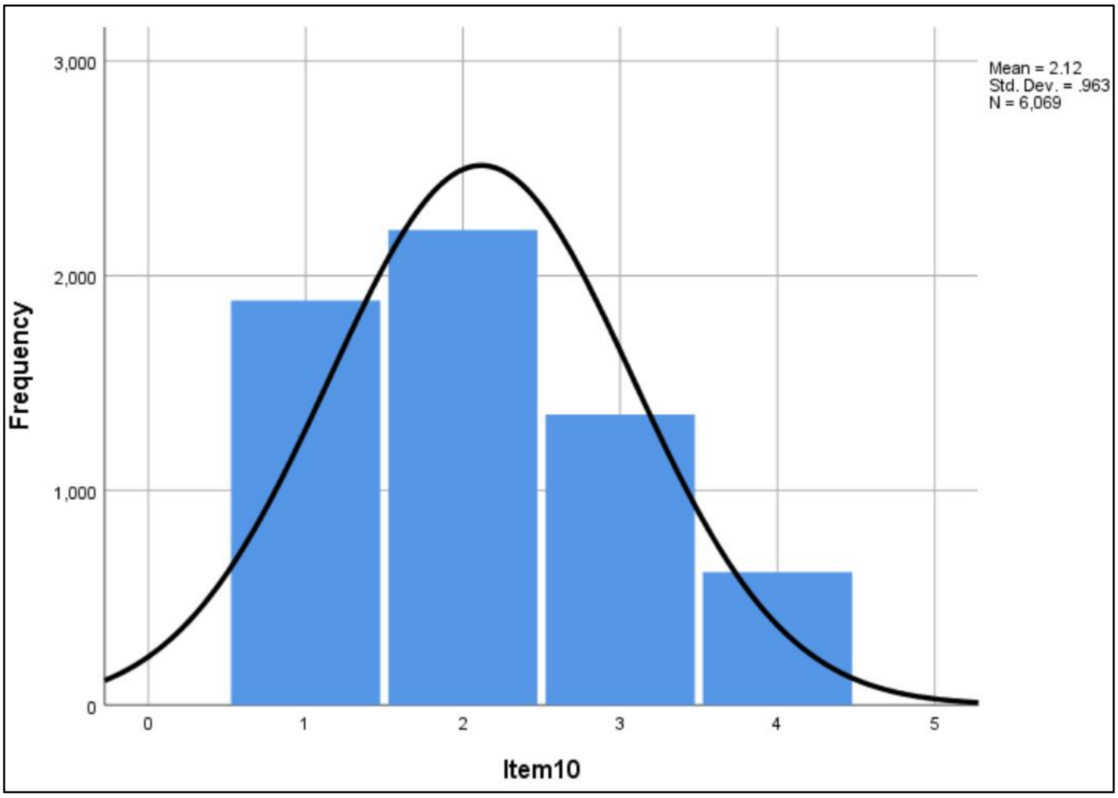

Figure S9 Distribution of scores in the item 10.

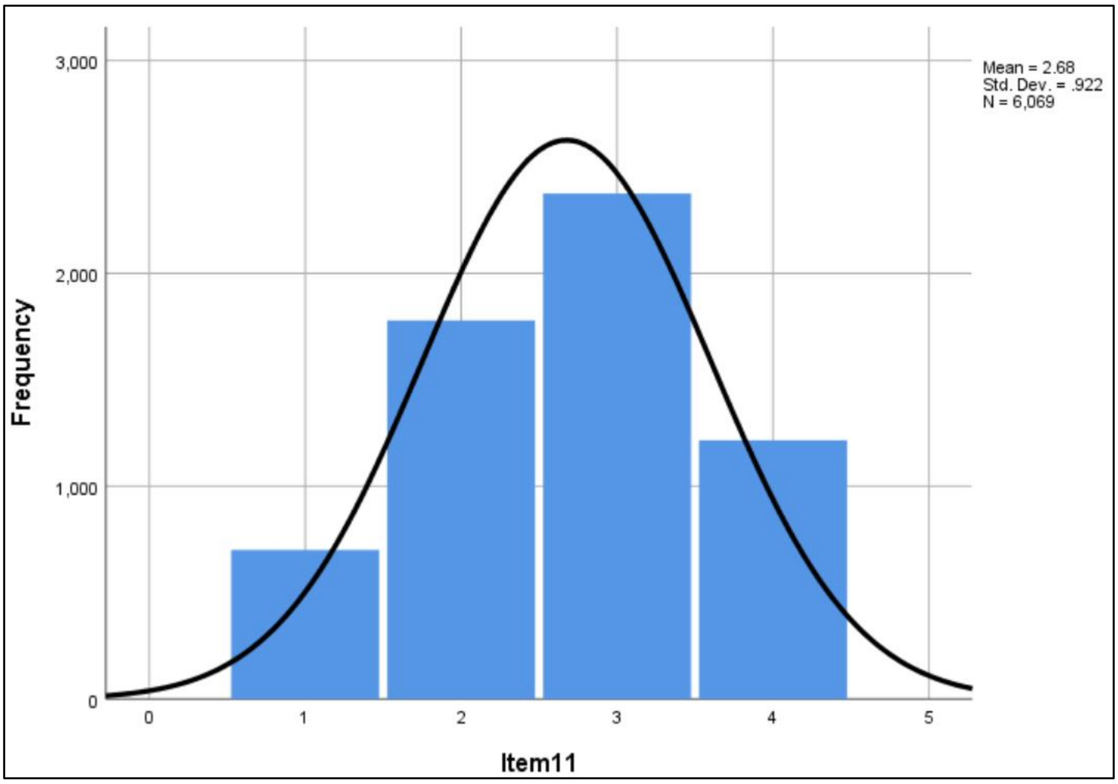

Figure S10 Distribution of scores in the item 11.

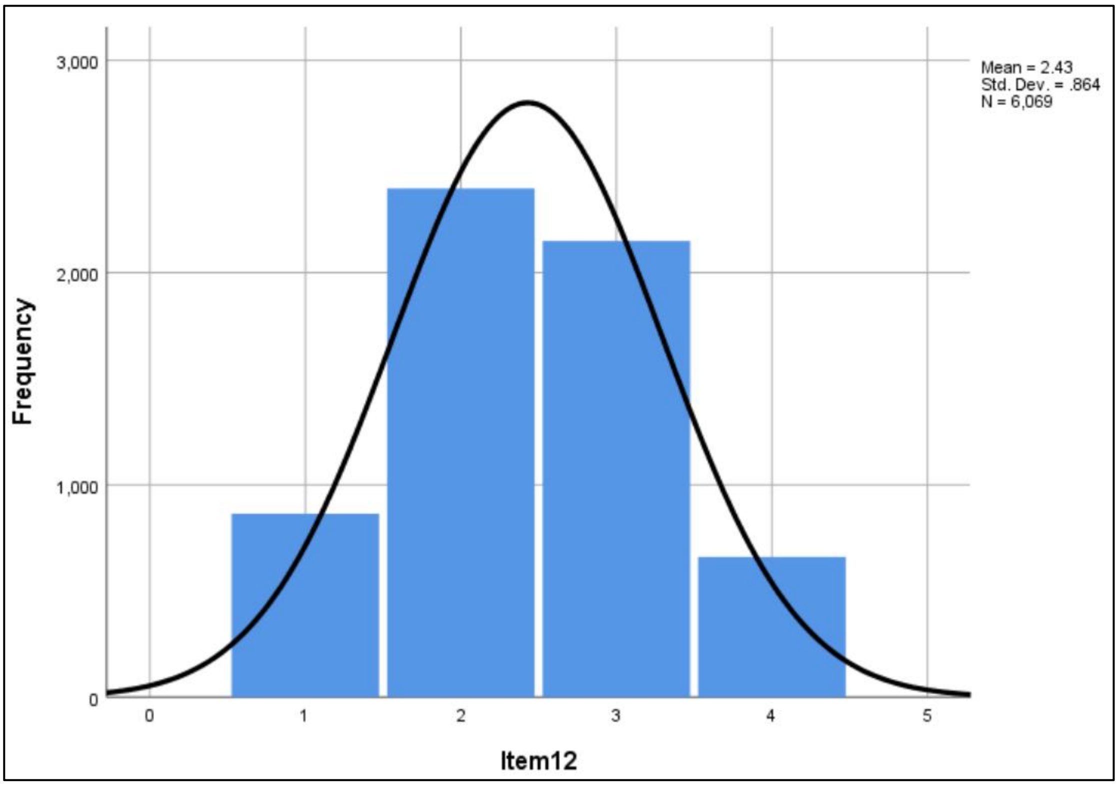

Figure S11 Distribution of scores in the item 12.

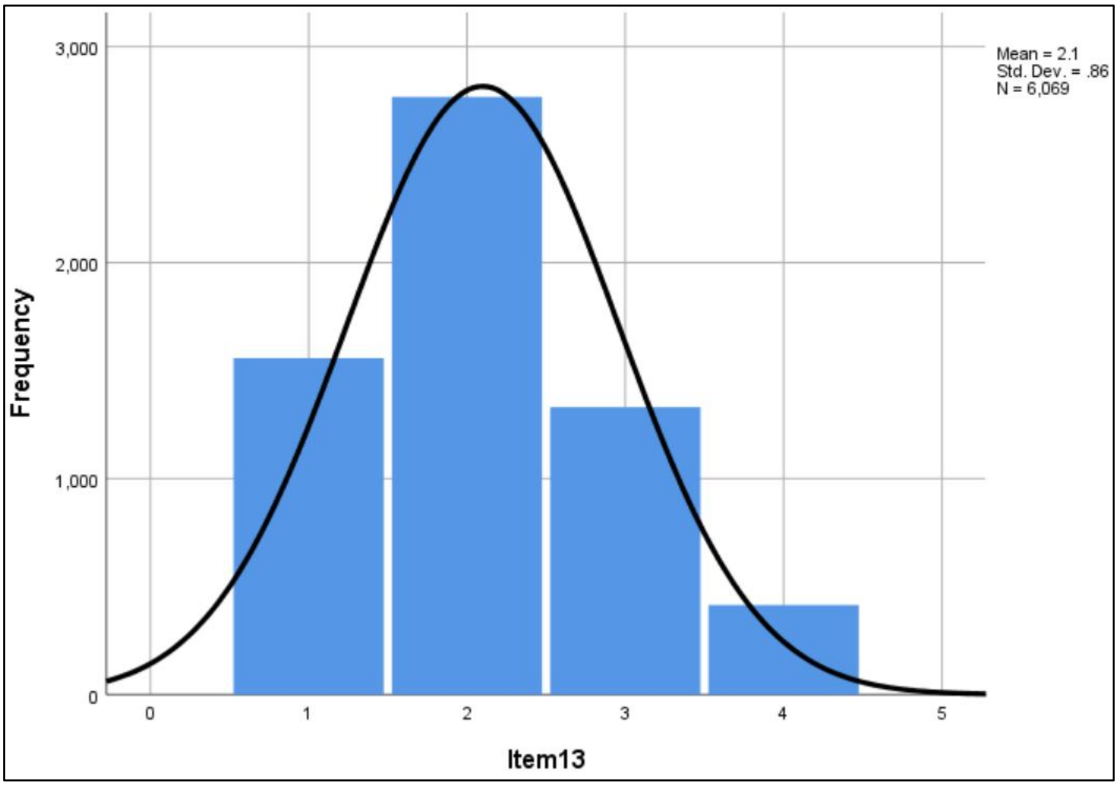

Figure S12 Distribution of scores in the item 13.

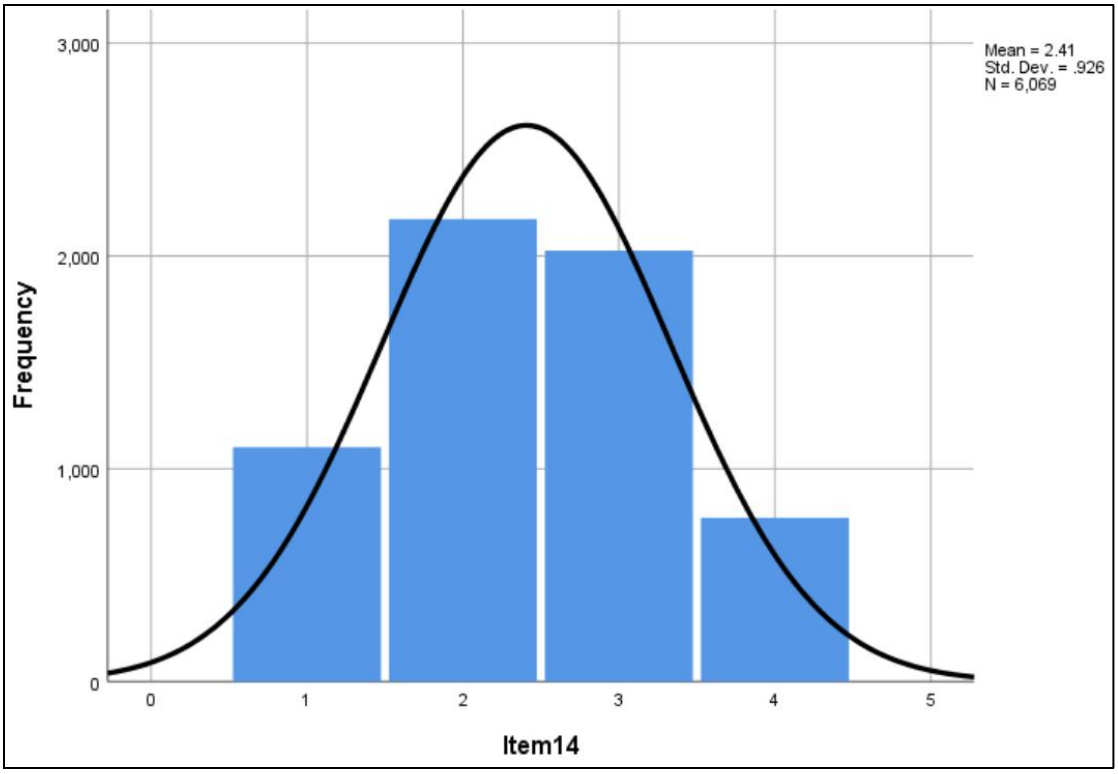

Figure S13 Distribution of scores in the item 14.

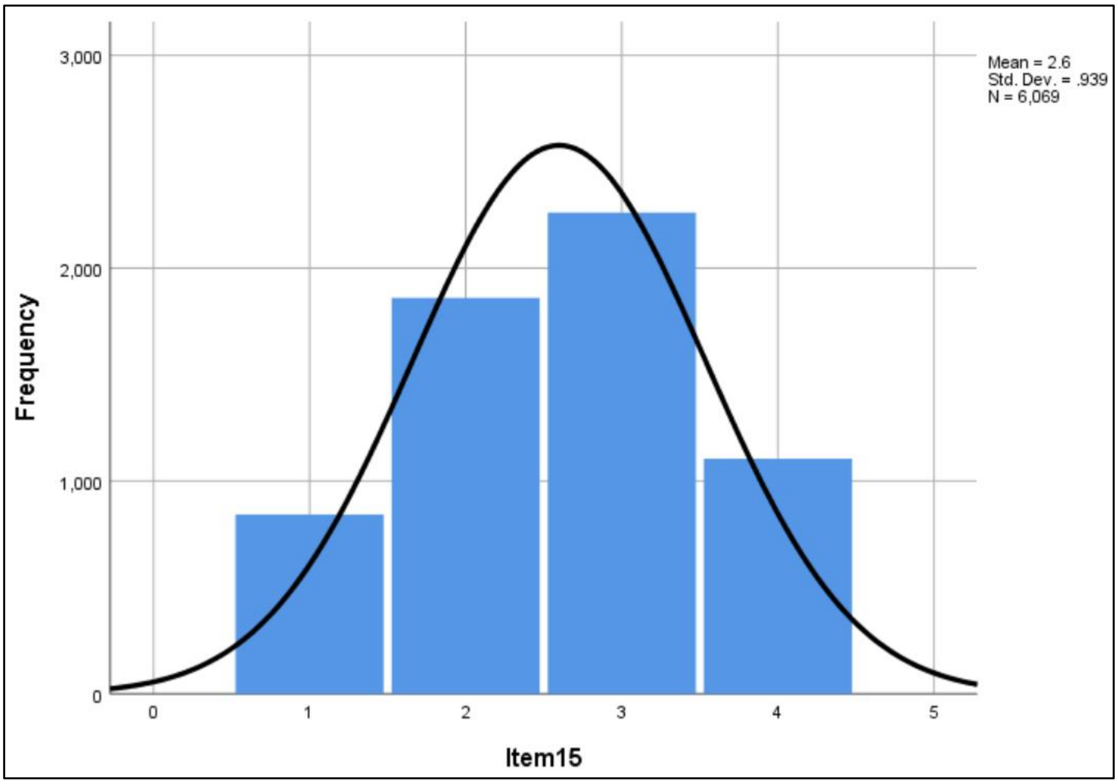

Figure S14 Distribution of scores in the item 15.

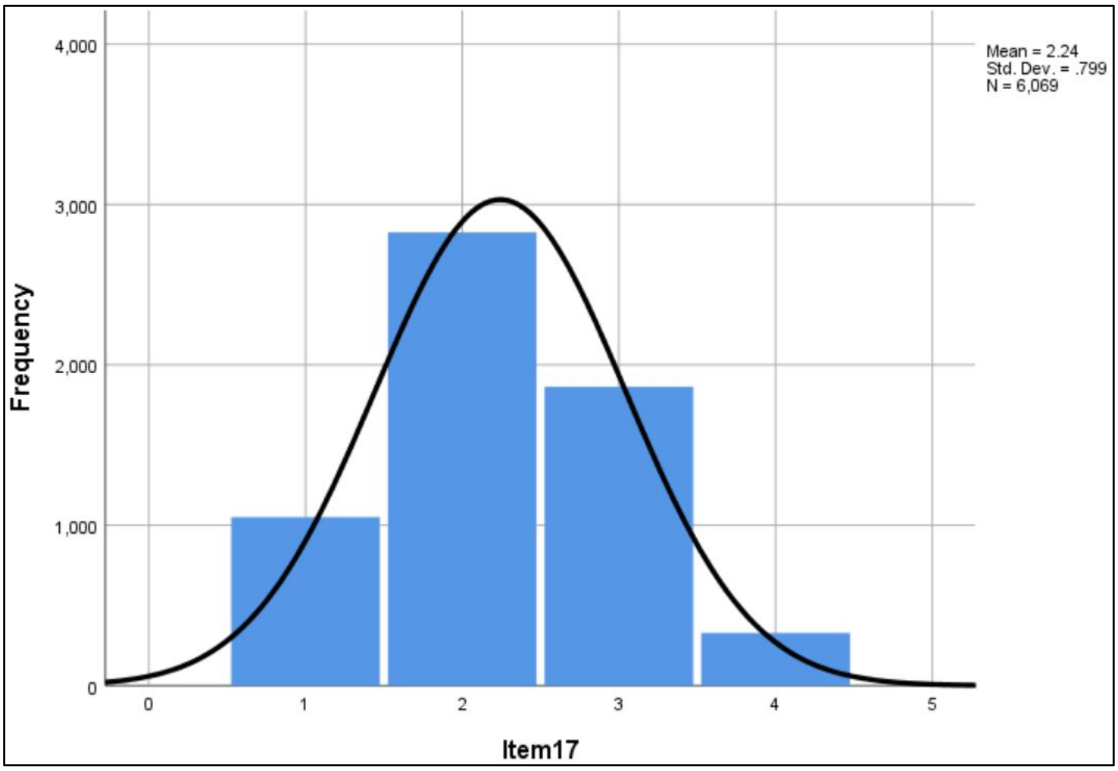

Figure S15 Distribution of scores in the item 17.

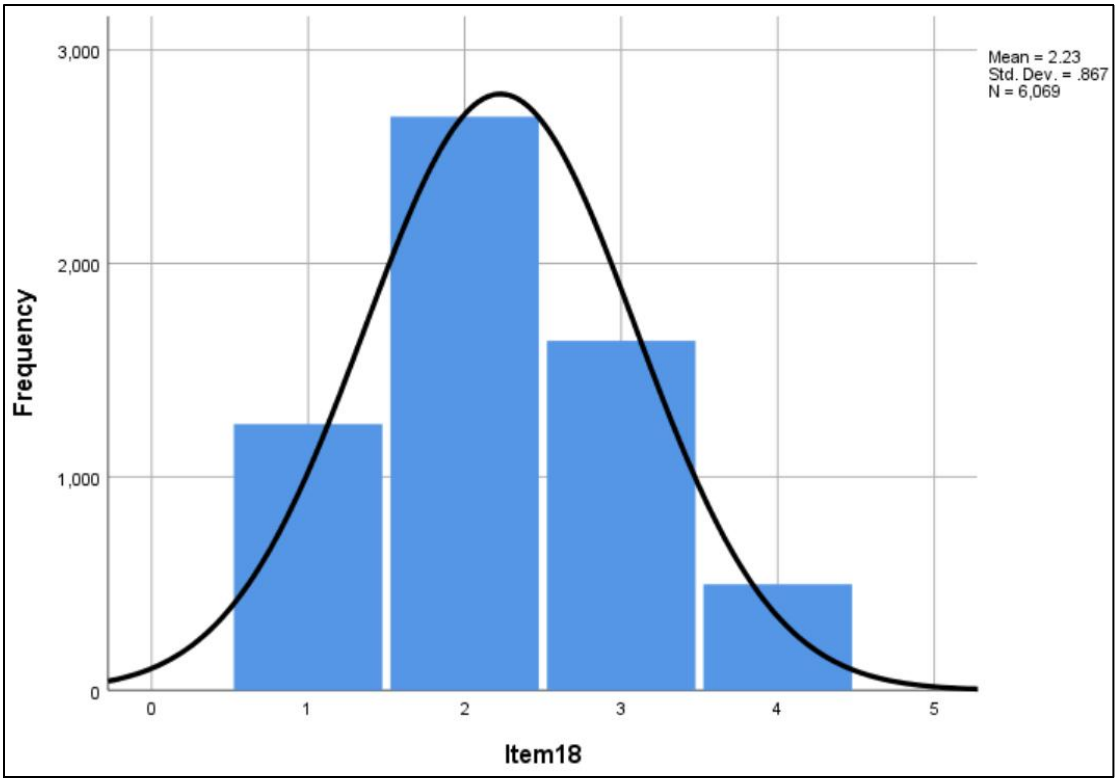

Figure S16 Distribution of scores in the item 18.

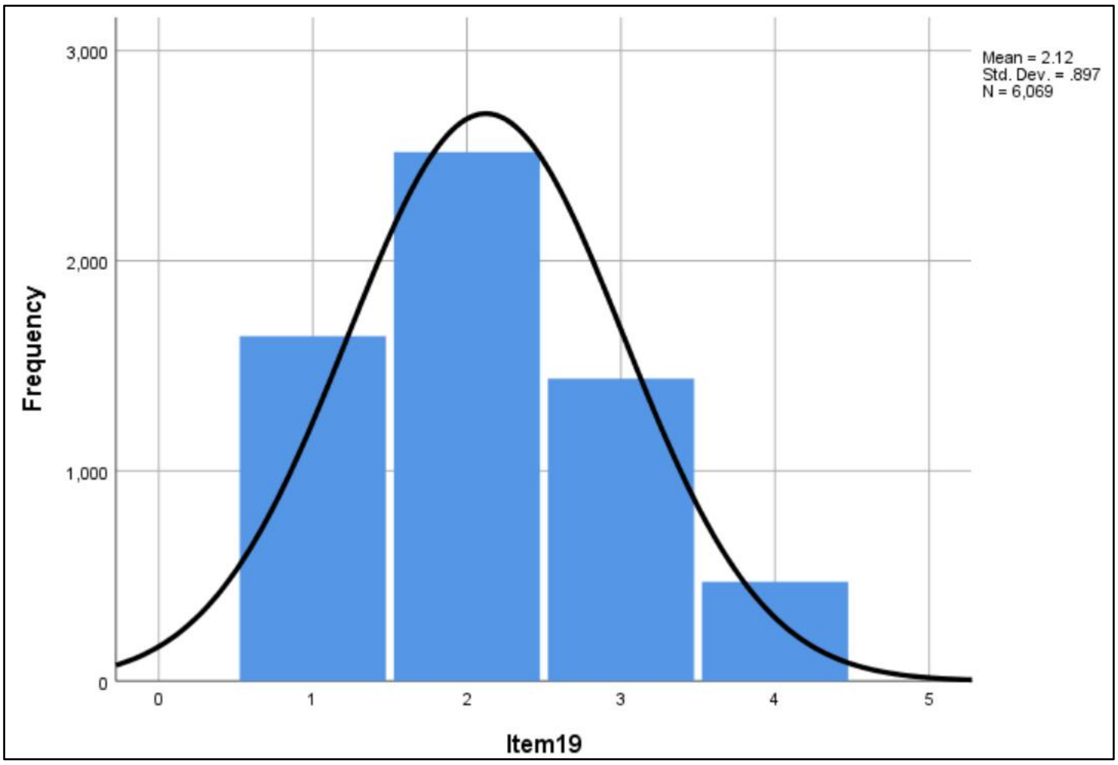

Figure S17 Distribution of scores in the item 19.

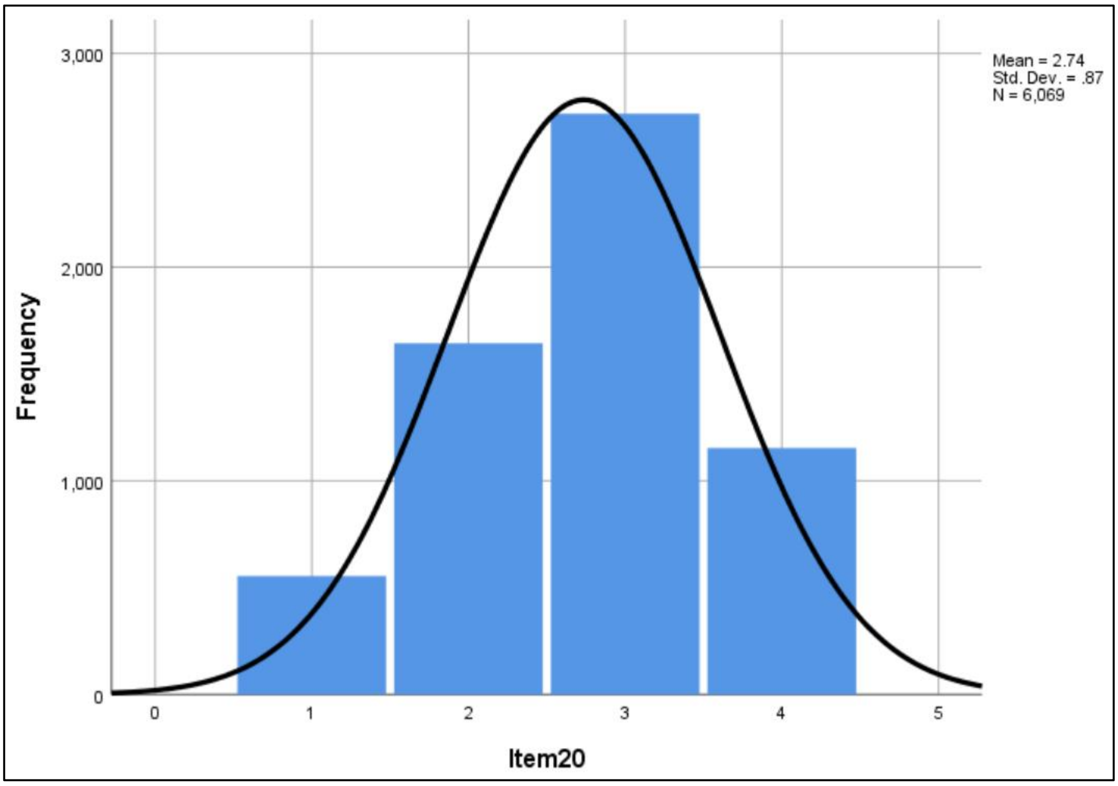

Figure S18 Distribution of scores in the item 20.

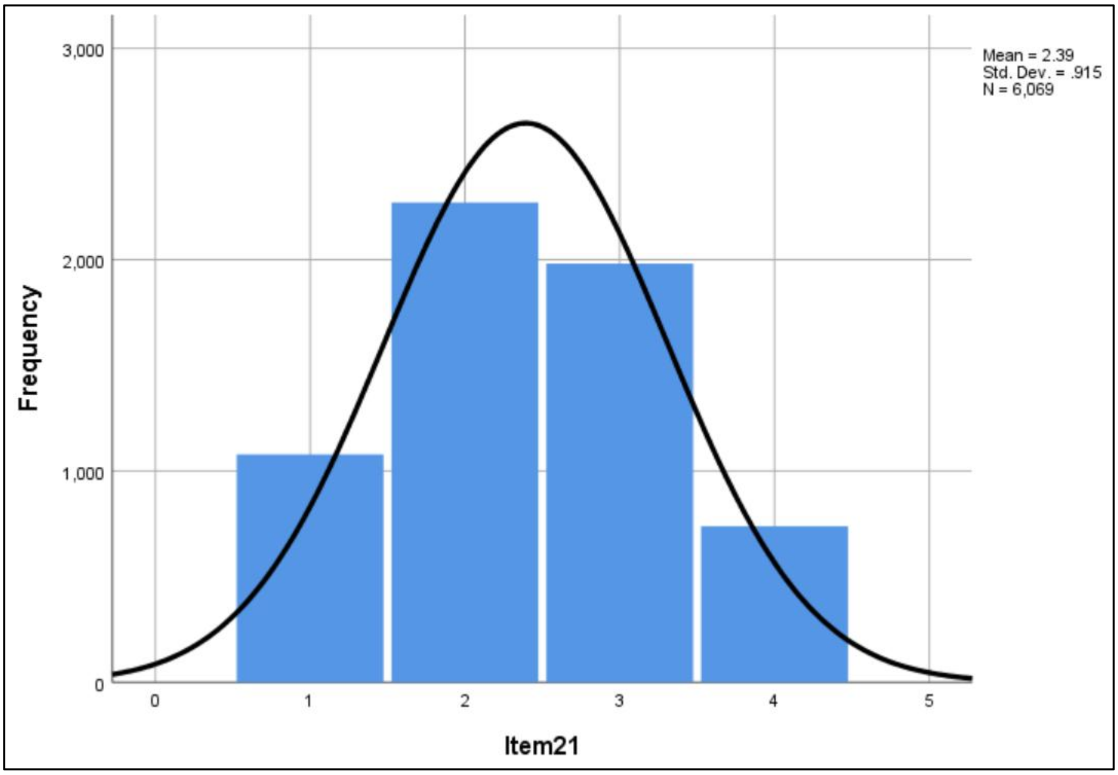

Figure S19 Distribution of scores in the item 21.

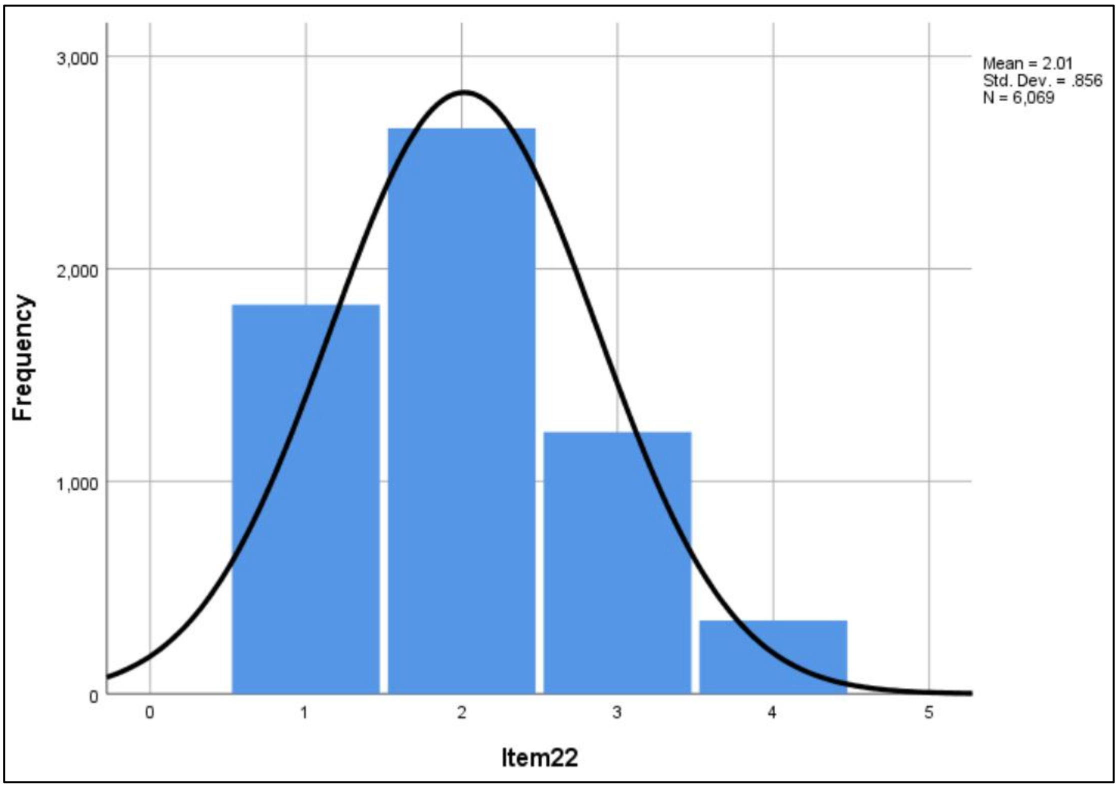

Figure S20 Distribution of scores in the item 22.

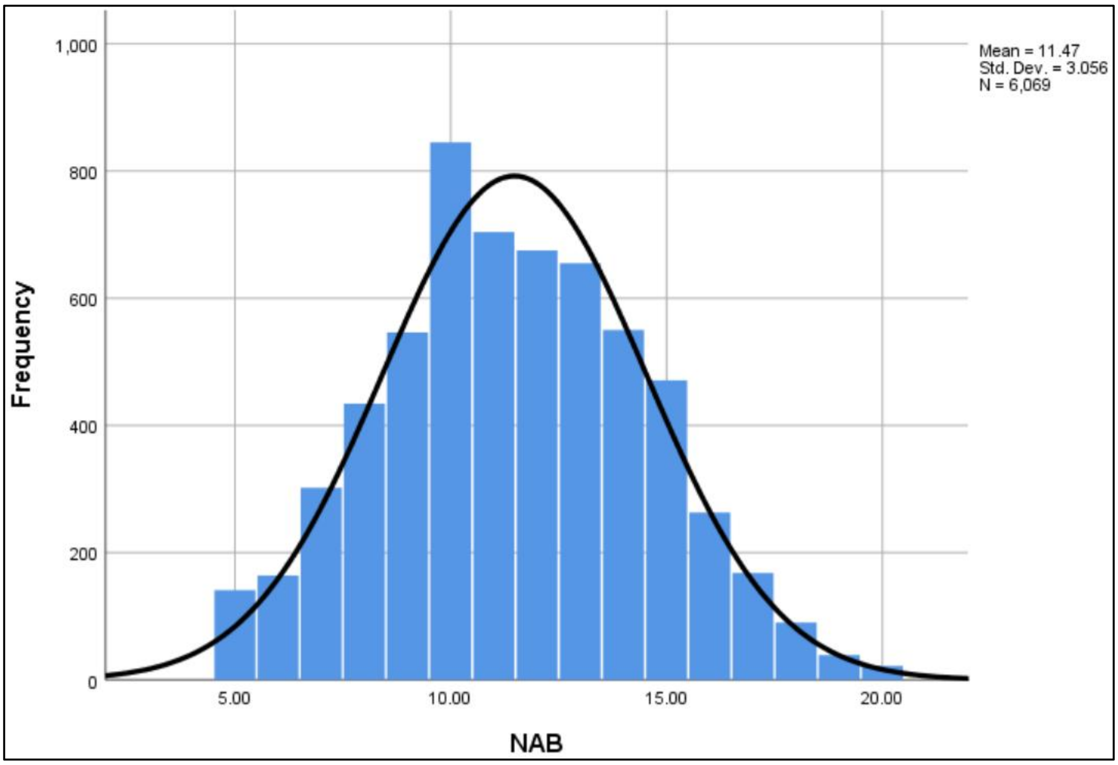

Figure S21 Distribution of scores in Negative Attention Bias (NAB).

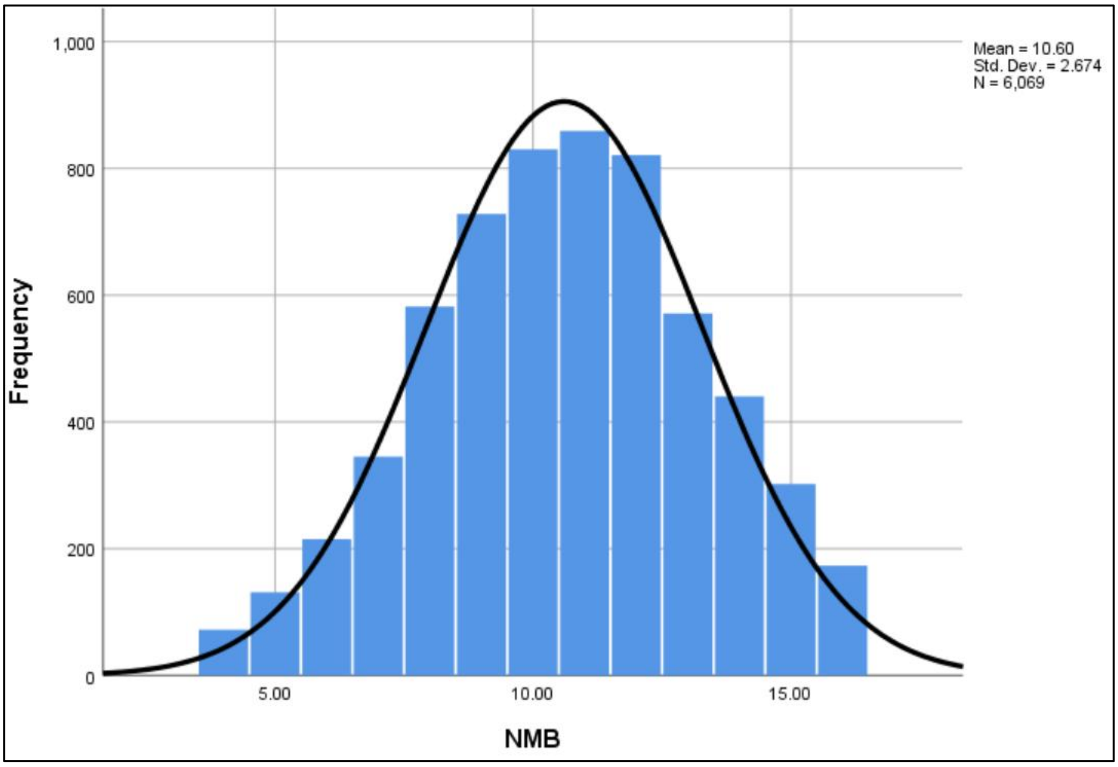

Figure S22 Distribution of scores in Negative Memory Bias (NMB).

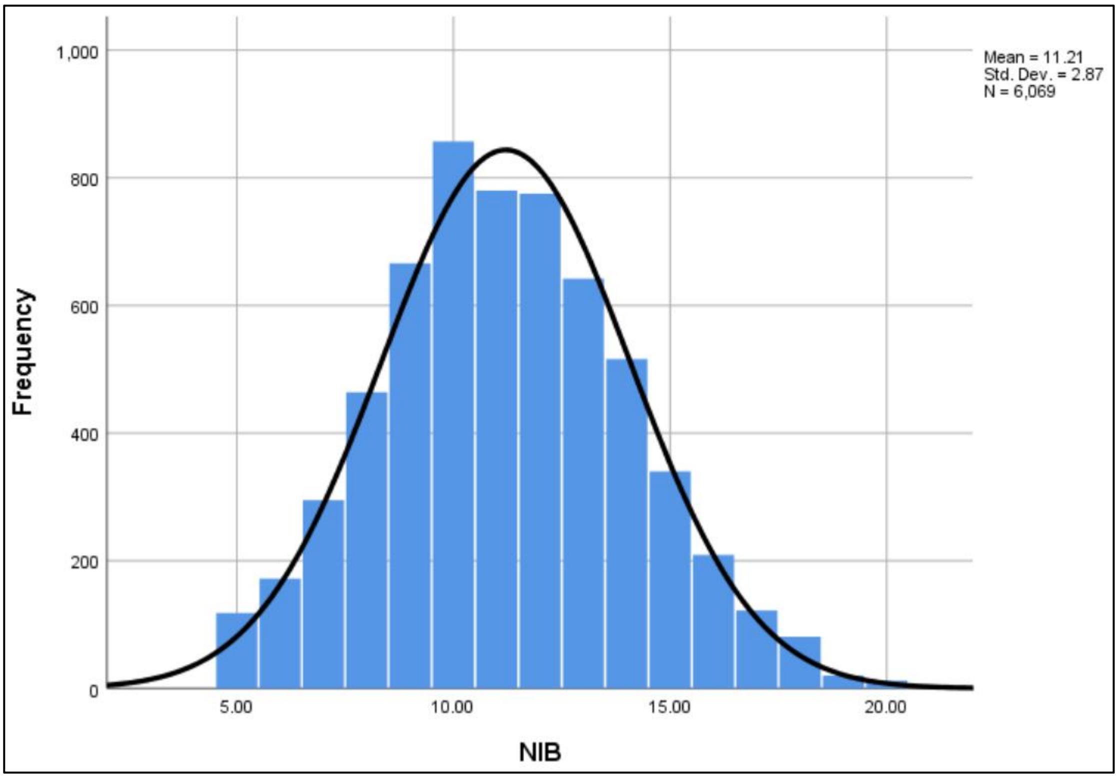

Figure S23 Distribution of scores in Negative Interpretation Bias (NIB).

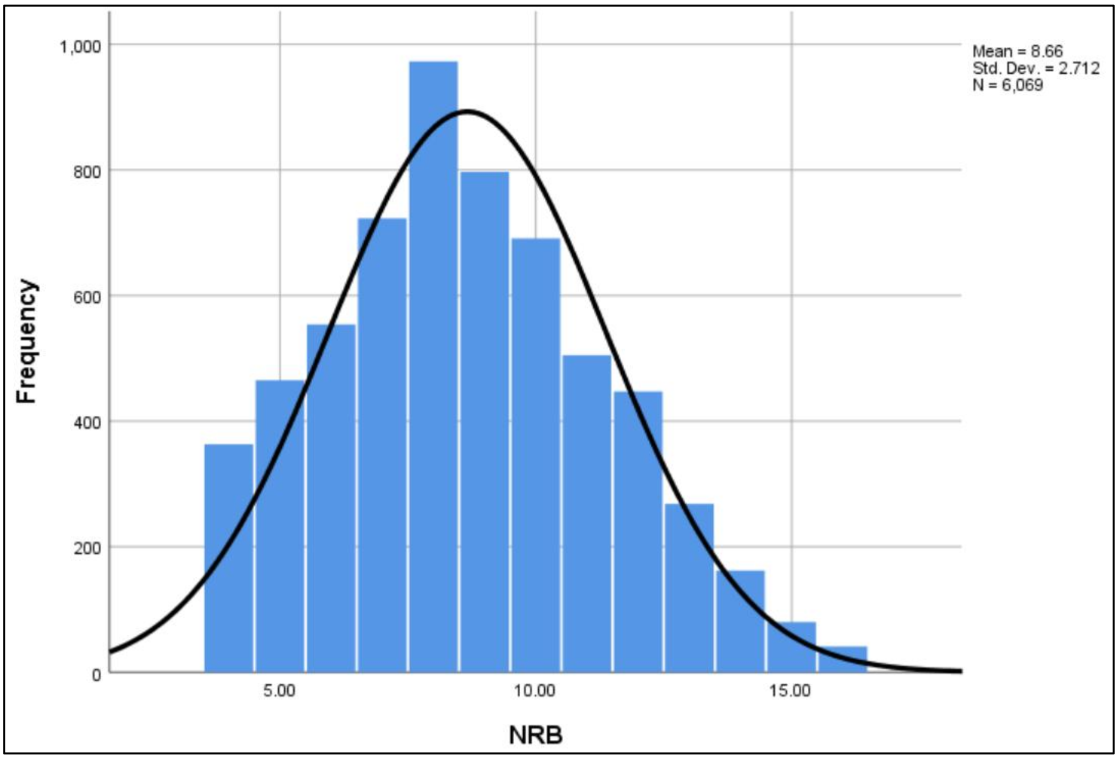

Figure S24 Distribution of scores in Negative Rumination Bias (NRB).

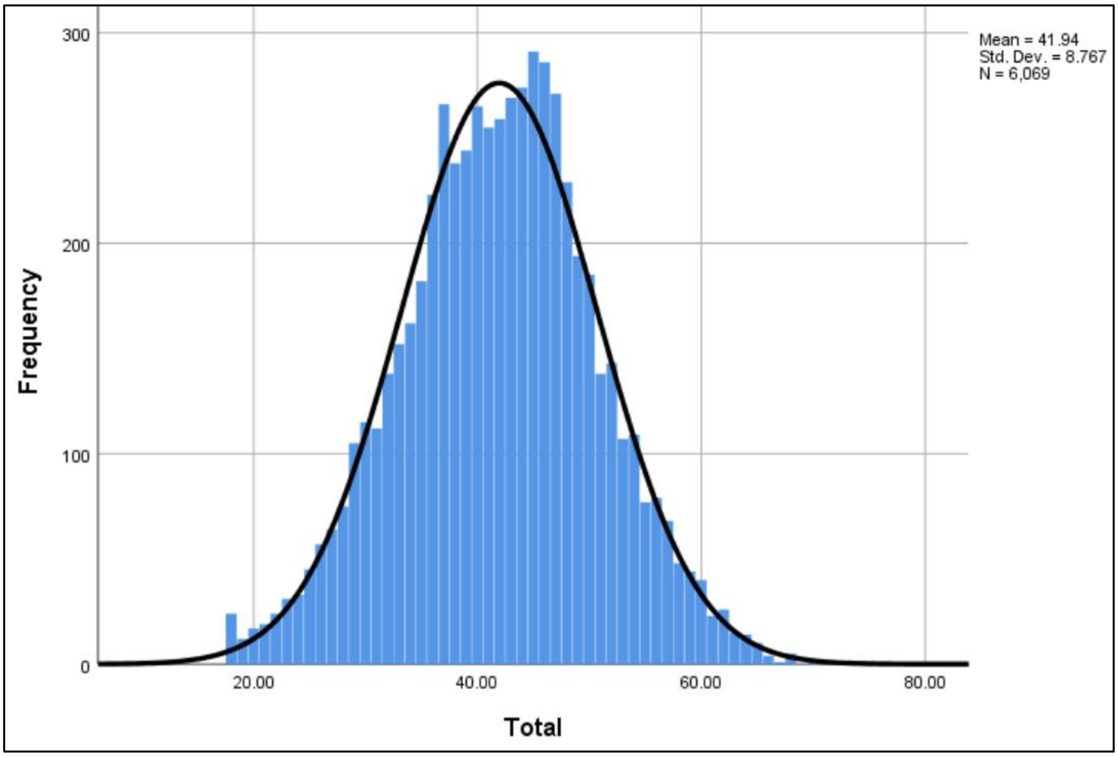

Figure S25 Distribution of scores in total scale.
